# Reproductive factors and hormone receptor status among very young (<35 years) breast cancer patients

**DOI:** 10.18632/oncotarget.4698

**Published:** 2015-07-01

**Authors:** Xiaoqing Jia, Guangyu Liu, Miao Mo, Jingyi Cheng, Zhenzhou Shen, Zhimin Shao

**Affiliations:** ^1^ Department of Breast Surgery, Key Laboratory of Breast Cancer in Shanghai, Fudan University Shanghai Cancer Center, Department of Oncology, Shanghai Medical College, Fudan University, Shanghai, P. R. China; ^2^ Clinical Statistics Center, Fudan University Shanghai Cancer Center, Department of Oncology, Shanghai Medical College, Fudan University, Shanghai, P. R. China; ^3^ Department of Nuclear Medicine, Fudan University Shanghai Cancer Center, Department of Oncology, Shanghai Medical College, Fudan University, Shanghai, P. R. China

**Keywords:** reproductive factors, menopause, parity, oral contraceptive, breast cancer

## Abstract

The prognosis for breast cancer occurs in young women is usually poor. The impact of different reproductive factors on disease characteristics is still largely unknown. We analyzed 261 patients aged ≤35 years old who were treated at the Cancer Hospital of Fudan University, Shanghai, China. The relationships between certain reproductive factors (age at menarche, parity, number of children, breastfeeding, history of abortion, age at first full-term pregnancy and oral contraceptive (OC) use) and disease characteristics were evaluated. Compared with patients who experienced fewer full-term pregnancies (<2 times), the patients with more full-term pregnancies (≥2 times) exhibited higher percentage of ER-positive tumors (61.5%) (*P* = 0.015), and patients whose age of menarche was ≥15 years exhibited a greater chance of PR-positive tumors (64.8%) (*P* = 0.036) compared with those whose age of menarche was <15 years old. Additionally, patients who had taken OCs were more likely to present with late-stage tumors (II stage or later) (87.5%) (*P* = 0.002) than patients who had never taken OCs. Our study provides evidence that women with more full-term pregnancies and later age at menarche are more possible to exhibit hormone receptor-positive tumors. Additionally, patients who have taken OCs are more likely to present with advanced disease.

## INTRODUCTION

Breast cancer rarely occurs in very young women (<35 years old), with this subgroup accounting for approximately 2% of all breast cancer patients at diagnosis in western countries [1]. Breast cancers that occur at a young age are typically less differentiated; exhibit more aggressive biological behavior, a higher grade, and a more advanced stage; and are associated with a more unfavorable prognosis [2–5]. Young patients have higher risks of local recurrence and distant metastasis compared with their older counterparts [6, 7]. Age younger than 35 years is an independent prognostic factor for progression free survival (PFS) [6]. Young breast cancer patients exhibit more aggressive disease characteristics and have a poorer prognosis [8]. The major clinical sub-classification of breast tumors is based on estrogen (ER) and progesterone (PR) receptor expressions, and this classification guides targeted therapies and provides important prognostic information [9]. In addition, recent epidemiological data indicate that the association between a woman's reproductive history and breast cancer differs based on ER and PR expressions [10]

Reproductive factors, such as age at menarche, age at first childbirth, time between menarche and first childbirth, number of children, oral contraceptive (OC) use and breastfeeding, have been suggested to be associated with an increased risk for breast cancer [10]. Therefore, this study aimed to investigate the relationships between the above mentioned reproductive factors and certain biological characteristics in a group of very young patients (<35 years of age) with operable breast cancer.

## RESULTS

### Baseline characteristics

A total of 359 patients aged ≤35 years and had breast cancer were identified. After excluding 74 patients who presented with non-invasive breast cancer and14 who presented with bilateral tumors, a total of 261 patients were included in the analysis. The patient characteristics are provided in Table 1.

**Table 1:** Biological factors distribution in the evaluable patients

Characteristic	No.of patients(%)
**Age (median, range)**	32(13-35)
**≤25**	10(3.8)
**26-30**	81(31)
**31-35**	170(65.2)
**Age at menarche (median, range)**	14(7-18)
**Age at first full-term pregnancy (median, range)**	25(18-33)
**Duration of breastfeeding(months)(median, range)**	11(0-99)
**Abortions**	
**0**	104(39.8)
**1**	50(19.2)
**2**	36(13.8)
**3**	11(4.2)
**4**	0(0)
**5**	1(0.4)
**Unknown**	59(22.6)
**Number of full-term pregnancies**	
**0**	21(8)
**1**	173(66.3)
**2**	25(9.6)
**3**	1(0.4)
**Unknown**	41(15.7)
**Oral contraceptive (OC) use**	
**Never OC user**	253(96.9)
**Past OC user**	8(3.1)
**Lymph node status**	
**Positive**	154(59)
**Negative**	79(30.3)
**Unknown**	28(10.7)
**Grade**	
**I**	0(0)
**II**	200(76.6)
**III**	61(23.4)
**Stage**	
**I**	63(24.1)
**II**	170(65.1)
**III**	26(10)
**IV**	2(0.8)
**ER status**	
**Positive**	140(53.6)
**Negative**	121(46.4)
**PR status**	
**Positive**	141(54)
**Negative**	120(46)
**HER2/neu status**	
**Positive**	65(24.9)
**Negative**	196(75.1)

### Impact of full-term pregnancy on biological characteristics

**Figure 1:**
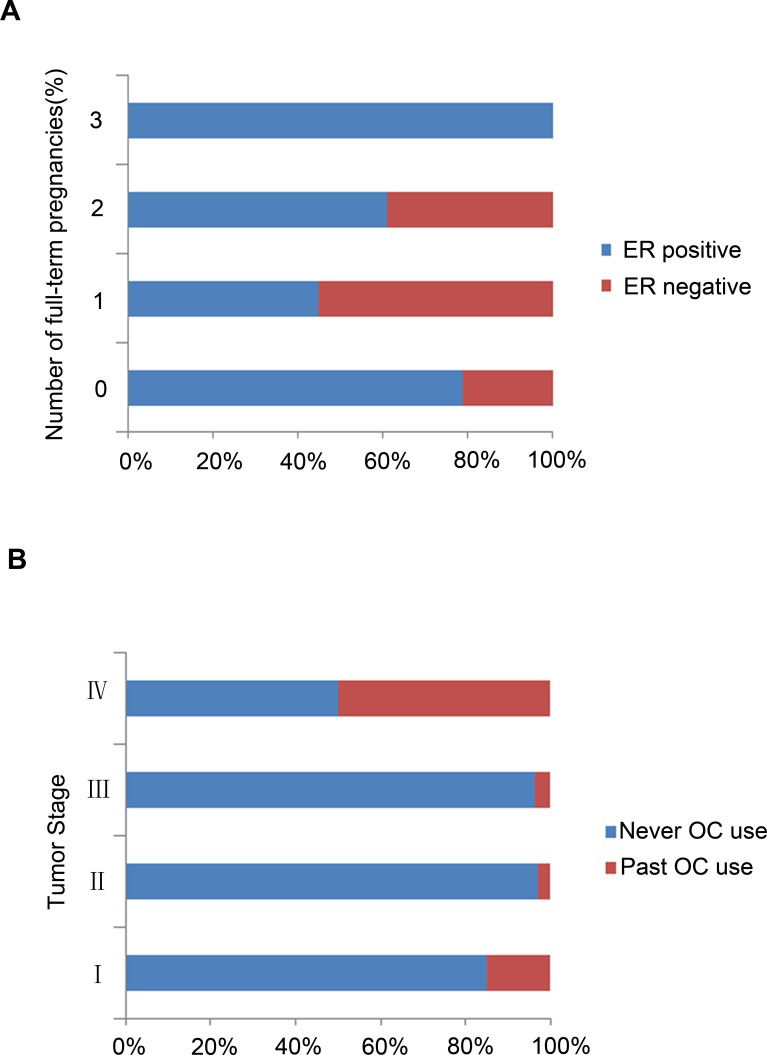
Hormone receptor (HR) expression levels in different breast cancer subgroups **A.** ER expression in the different full-term pregnancy breast cancer subgroups; **B.** PR expression stratified by OC use.

We compared the characteristics of women with different numbers of full-term pregnancies to study the impact of pregnancy on the development of breast cancer at a very young age (Table 2). There was an increased percentage of ER-positive tumors in the group with more full-term pregnancies compared with the group with few full-term pregnancies (*P* = 0.015). No differences were noted between the number of full-term pregnancies and the number of involved axillary lymph nodes, tumor grade, TNM (tumor, node, metastasis) stage, lymph node metastasis, PR or HER2/neu expression. ER expression in the different full-term pregnancy subgroups are presented in Figure 1A.

**Table 2:** Impact of full-term pregnancy on disease characteristics

	Number of full-term pregnancies No.of patients(%)				*P* value
	0	1	2	3	
**Age**					0.037
**≤25**	2(33.3)	4(66.7)	0(0)	0(0)	
**26-30**	9(16.1)	45(80.4)	2(3.5)	0(0)	
**31-35**	10(6.3)	124(78.5)	23(14.6)	1(0.6)	
**Lymph node status**					0.583
**Positive**	10(7.5)	109(82)	13(9.8)	1(0.7)	
**Negative**	6(9.1)	50(75.8)	10(15.1)	0(0)	
**Grade**					0.826
**I**	0(0)	0(0)	0(0)	0(0)	
**II**	17(10)	135(79)	18(10.5)	1(0.5)	
**III**	4(8.2)	38(77.6)	7(14.2)	0(0)	
**Stage**					0.222
**I**	7(13.7)	39(76.5)	5(9.8)	0(0)	
**II**	13(8.9)	116(79.5)	17(11.6)	0(0)	
**III**	1(4.8)	16(76.2)	3(14.3)	1(4.7)	
**IV**	0(0)	2(100)	0(0)	0(0)	
**ER status**					0.015
**Positive**	17(14.5)	83(70.9)	16(13.7)	1(0.9)	
**Negative**	4(3.9)	90(87.4)	9(8.7)	0(0)	
**PR status**					0.207
**Positive**	14(11.6)	89(73.6)	17(14)	1(0.8)	
**Negative**	7(7.1)	84(84.8)	8(8.1)	0(0)	
**HER2/neu status**					0.401
**Positive**	5(8.5)	47(79.7)	6(10.2)	1(1.6)	
**Negative**	16(9.9)	126(78.3)	19(11.8)	0(0)	

### Impact of abortion on biological characteristics

We next determined whether the number of abortions had an impact on hormone receptor (HR) expression (Table 3). In contrast to the profound impact of the number of full-term pregnancies, no distinct association was noted between the number of abortions and disease characteristics, and no significant differences were detected in terms of node status, tumor grade, TNM stage, or the expression of ER, PR or HER2/neu.

**Table 3:** Impact of abortion on disease characteristics

	Abortions No.of patients (%)					*P* value
	0	1	2	3	5	
**Age**						0.9
**≤25**	3(50)	2(33.3)	1(17.7)	0(0)	0(0)	
**26-30**	28(58.3)	12(25)	5(10.4)	3(6.3)	0(0)	
**31-35**	73(49.3)	36(24.3)	30(20.3)	8(5.4)	1(0.67)	
**Lymph node status**						0.513
**Positive**	58(47.9)	32(26.4)	24(19.8)	6(5)	1(0.9)	
**Negative**	40(60.6)	14(21.2)	9(13.6)	3(4.6)	0(0)	
**Grade**						0.953
**I**	0(0)	0(0)	0(0)	0(0)	0(0)	
**II**	80(51)	38(24.2)	29(18.5)	9(5.7)	1(0.6)	
**III**	24(53.3)	12(26.7)	7(15.6)	2(4.4)	0(0)	
**Stage**						0.922
**I**	24(52.2)	10(21.7)	11(23.9)	1(2.2)	0(0)	
**II**	68(50.7)	36(26.9)	20(14.9)	9(6.7)	1(0.8)	
**III**	11(55)	4(20)	4(20)	1(5)	0(0)	
**IV**	1(50)	0(0)	1(50)	0(0)	0(0)	
**ER status**						0.777
**Positive**	55(51.9)	28(26.4)	17(16)	5(4.7)	1(1)	
**Negative**	49(51)	22(22.9)	19(19.8)	6(6.3)	0(0)	
**PR status**						0.566
**Positive**	57(52.8)	24(22.2)	22(20.4)	5(4.6)	0(0)	
**Negative**	47(50)	26(27.7)	14(14.9)	6(6.4)	1(1)	
**HER2/neu status**						0.484
**Positive**	26(53.1)	12(24.5)	8(16.3)	2(4.1)	1(2)	
**Negative**	78(51)	38(24.8)	28(18.3)	9(5.9)	0(0)	

### Impact of age at menarche on biological characteristics

Next, we analyzed the age at menarche among very young breast cancer patients. There was a significantly greater percentage of PR-positive tumors among patients whose age at menarche was ≥15 years compared with those whose age at menarche was <15 years (*P* = 0.036). Age at menarche was not significantly associated with the number of involved axillary lymph nodes, tumor grade, TNM stage, lymph node metastasis, or the expression of ER or HER2/neu (Table 4).

**Table 4:** Impact of menarcheal age on disease characteristics

	Menarcheal age (years) No.of patients(%)			*P* value
	≤12	13-14	≥15	
**Age**				0.531
**≤25**	2(20)	6(60)	2(20)	
**26-30**	12(15.6)	40(51.9)	25(32.5)	
**31-35**	16(9.9)	85(52.5)	61(37.7)	
**Lymph node status**				0.742
**Positive**	20(13.4)	77(51.7)	52(34.9)	
**Negative**	8(10.8)	42(56.8)	24(32.4)	
**Grade**				0.818
**I**	0(0)	0(0)	0(0)	
**II**	22(11.6)	99(52.1)	69(36.3)	
**III**	8(13.6)	32(54.2)	19(32.2)	
**Stage**				0.304
**I**	4(6.8)	37(62.7)	18(30.5)	
**II**	23(14.1)	84(51.5)	56(34.4)	
**III**	3(12)	9(36)	13(52)	
**IV**	0	1(50)	1(50)	
**ER status**				0.281
**Positive**	15(11.3)	65(48.9)	53(39.8)	
**Negative**	15(12.9)	66(56.9)	35(14.1)	
**PR status**				0.036
**Positive**	15(11.2)	62(46.3)	57(42.5)	
**Negative**	15(13)	69(60)	31(27)	
**HER2/neu status**				0.071
**Positive**	6(9.5)	41(65.1)	16(25.4)	
**Negative**	24(12.9)	90(48.4)	72(38.7)	

### Impact of age at first full-term childbirth and the oral contraceptive use on biological characteristics

We compared the characteristics of very young breast cancer patients with different ages at first full-term childbirth (Table 5). No distinct associations were noted between age at the first full-term birth and disease characteristics, and no significant differences were detected regarding node status, tumor grade, TNM stage, expression of ER, PR or HER2/neu.

**Table 5:** Impact of age at first full-term childbirth on disease characteristics

	Age at first full-term childbirth (years) No.of patients(%)				*P* value
	≤19	20-24	25-29	30-35	
**Age**					0.025
**≤25**	0(0)	3(75)	1(25)	0(0)	
**26-30**	3(8.3)	8(22.2)	25(69.4)	0(0)	
**31-35**	1(0.8)	43(33.9)	74(58.3)	9(7.1)	
**Lymph node status**					0.51
**Positive**	3(3)	34(34)	57(57)	6(6)	
**Negative**	1(1.8)	13(23.6)	38(69.1)	3(5.5)	
**Grade**					0.485
**I**	0(0)	0(0)	0(0)	0(0)	
**II**	4(3)	40(30.3)	80(60.6)	8(6.1)	
**III**	0(0)	14(40)	20(57.1)	1(2.9)	
**Stage**					0.845
**I**	1(2.7)	9(24.3)	26(70.3)	1(2.7)	
**II**	3(2.8)	35(32.1)	64(58.7)	7(6.4)	
**III**	0(0)	9(47.4)	9(47.4)	1(5.3)	
**IV**	0(0)	1(50)	1(50)	0(0)	
**ER status**					0.739
**Positive**	1(1.2)	25(30.9)	50(61.7)	5(6.2)	
**Negative**	3(3.5)	29(33.7)	50(61.7)	4(4.7)	
**PR status**					0.433
**Positive**	2(2.3)	32(37.2)	49(57)	3(3.5)	
**Negative**	2(2.5)	22(27.2)	51(63)	6(7.4)	
**HER2/neu status**					0.329
**Positive**	1(2.6)	11(28.9)	26(68.4)	0(0)	
**Negative**	3(2.3)	43(33.3)	74(57.4)	9(7)	

We also analyzed OC use among very young breast cancer patients. Patients who had taken OCs were more likely to present with late-stage tumors (*P* = 0.002) than those who had never taken OCs (Table 6). No distinct association was noted between OC use and disease characteristics, and no significant differences were identified in node status, tumor grade, expression of ER, PR or HER2/neu. PR expression stratified by OC use is presented in Figure 1B.

**Table 6:** Impact of oral contraceptive use on disease characteristics

	OC use No.of patients(%)		*P* value
	Never OC use	Past OC use	
**Age**			0.422
**≤25**	9(90)	1(10)	
**26-30**	79(97.5)	2(2.5)	
**31-35**	165(97.1)	5(2.9)	
**Lymph node status**			0.266
**Positive**	148(96.1)	6(3.9)	
**Negative**	78(98.7)	1(1.3)	
**Grade**			0.912
**I**	0(0)	0(0)	
**II**	194(97)	6(3)	
**III**	59(96.7)	2(3.3)	
**Stage**			0.002
**I**	62(98.4)	1(1.6)	
**II**	165(97.1)	5(2.9)	
**III**	25(96.2)	1(3.8)	
**IV**	1(50)	1(50)	
**ER status**			0.051
**Positive**	133(95)	7(5)	
**Negative**	120(99.2)	1(0.2)	
**PR status**			0.054
**Positive**	134(95)	7(5)	
**Negative**	119(99.2)	1(0.2)	
**HER2/neu status**			0.995
**Positive**	63(96.9)	2(3.1)	
**Negative**	190(96.9)	6(3.1)	

## DISCUSSION

Very young women with breast cancer present with aggressive disease and have a worse prognosis than their older counterparts. One study in our department has indicated that age was an independent predictive factor for relapse free survival (RFS). Breast cancer patients <or = 40 years presented more unfavourable features than those 41-50 years patients [11]. Our study identified a significant association between the number of full-term pregnancies and age at menarche with tumor HR status. Specifically, a greater number of full-term childbirths are associated with an increased percentage of ER-positive disease, whereas a later age at menarche is associated with an increased percentage of PR-positive disease among very young breast cancer patients. Additionally, patients who had taken OCs were more likely to present with late-stage tumors compared with those who had never taken OCs. Age at first full-term childbirth and the number of abortions were generally not associated with a HR positive or HR-negative breast cancer.

Several earlier studies indicated a strong association between older age at menarche or full-term childbirth (particularly, at an early age) and ER-positive breast cancer [3, 12–14]. Phipps et al. reported that even one full-term childbirth was associated with an increased risk for triple-negative breast cancer (ER-, PR- and HER2-), and the positive association was strengthened with an increasing number of full-term births [13]. Unfortunately, a consensus on the association of the number of full-term births, age at menarche and OC use with HR-positive breast cancer has not yet been achieved, especially for very young women (<35 years).

Previous studies investigating the association between different reproductive factors and ER-positive breast cancer have demonstrated a relatively consistent inverse risk association with full-term birth [3]. However, in our study, there was an increased percentage of ER-positive tumors among very young breast cancer patients with a greater number of full-term births (*P* = 0.015). The number of full-term births is considered to have a protective effect on breast cancer risk. Theoretically, this effect may be related to a more favorable breast cancer subtype (HR-positive).

We observed an association between increased age at menarche and PR expression. There was an increased percentage of PR-positive tumors among patients whose menarche age was ≥15 years compared with those whose age at menarche was <15 years old (*P* = 0.036). The inverse association between age at menarche and an increased breast cancer risk is thought to result from increased estrogen exposure during a woman's reproductive life [2]. We did not observe a relationship between the age at menarche and ER status. Women with a greater number of full-term childbirths and a later menarche age experienced an increased risk of harboring an endocrine-responsive tumor.

OC use has been extensively studied in numerous epidemiological studies, and the majority of these studies have reported either no association or a moderately increased risk for breast cancer, particularly among very young women and recent OC users [15]. Patients who had taken OCs were more likely to present with late-stage tumors (*P* = 0.002) compared with patients who had never taken OCs. In the current study, we were unable to confirm any significant risk associations between OC use and HR-positive breast cancer. Only a few baseline OC users were included in this cohort. In addition, OCs contain different estrogen and progestin types at varying concentrations [16], which could potentially mask any risk association.

We did not observe any association between age at first full-term childbirth or breastfeeding duration and the HR status or other biological characteristics, perhaps due to the lack of available data. At the time of this study, additional information on HER2 expression to classify breast cancer subtypes into more detailed molecular sub-classifications was not available. However, as routine HER2 testing is a relatively recent technique compared with ER and PR assessments, future cohort analyses will be able to include HER2 testing. In addition, further prospective studies with larger sample sizes are necessary.

Recently, Heidi et al [17] found that the expression and function of ER and PR in normal human breast is non-overlapping. ER and PR are functionally enriched in distinct cell subsets in the normal human breast. However, ER and PR become correlated in breast cancer and converge on common pathways. The effect of full-time pregnancies and age at menarche on the breast cancer hormone receptor status may result from that they influence different cell subsets which preferentially expressing ER and PR, respectively in normal human breast. The expression of hormone receptor and HER2 could be changed due to therapeutic reasons, such as neoadjuvant chemotherapy (NCT) [18]. Jin et al found that 13% of breast cancer patients changed from HR (+) to HR (−), 5.4% changed from HR (−) to HR (+) after NCT.

In conclusion, our study provides possible evidence that women with a greater number of full-term pregnancies or a later age at menarche have an increased risk of harboring an endocrine-responsive tumor. Additionally, patients who have taken OCs are more likely to present with advanced disease. Further studies that include more cases are needed to provide more precise risk estimations for reproductive factors and their associations with HR-positive tumors among very young women.

## PATIENTS AND METHODS

### Patients and follow-up

In total, 261 patients aged <35 years who underwent breast surgery between January 1, 1999, and December 31, 2007, were enrolled in this study. The study was approved by the ethics committee of the Cancer Hospital of Fudan University. Written informed consent was obtained from all of the patients prior to initiating any medical procedures. Patient information and clinicopathological characteristics were retrieved from the inpatient computerized database of the Cancer Hospital of Fudan University, Shanghai, China. A total of 261 young female patients (<35 years) without distant metastasis at first diagnosis were included in our study based on specific inclusion and exclusion criteria. Patients who were not eligible for surgery received 4 to 6 cycles of different neoadjuvant chemotherapy and/or hormone therapy (if required) according to the standard therapy at the time of surgery. The median age of this cohort was 32 years. The use of adjuvant radiotherapy was determined according to the database of the Cancer Hospital of Fudan University, Shanghai, China.

### Determination of biological characteristics

The immunohistochemical status of each sample was defined by staining for ER, PR and human epidermal growth factor 2 (HER2/neu). All of the primary monoclonal antibodies were provided by Dako (DAKO, Carpinteria, CA, USA) and were used at dilutions of 1:35 (ER), 1:50(PR) and 1:400(HER2/neu). HER2/neu overexpression was evidenced by complete membrane staining of at least 10% of the neoplastic cells. The percentage of cells with definite nuclear staining among at least 2000 neoplastic cells examined at 400× magnification was recorded when evaluating ER and PR status. The stained slides were evaluated independently by two of the authors.

### Pregnancy characteristics

Age at menarche (years) was categorized as ≤12, 13-14 and ≥15. The number of full-term pregnancies was categorized as 0, 1, 2, or 3. The age at first full-term childbirth (years) was defined as ≤19, 20-24, 25-29, or 30-35. The total cumulative breastfeeding duration (months) was categorized as ≤1, 2-6, 7-12, 13-17 or ≥18. OC use was categorized as never or past OC use.

This analysis aimed to analyze the relationship between tumor characteristics and biological markers in different groups of patients with various numbers of full-term pregnancies. Several of the main prognostic features were missing for several of the patients, and various factors were subsequently retrieved. HER2/neu staining was performed on a routine basis to assess its overexpression. Fluorescent in situ hybridization (FISH) was not routinely performed.

### Statistical analysis

The associations between categorical variables were measured using contingency tables with two-sided χ2 tests. All of the *P*-values were two-tailed, and the significance level was set at *p* < 0.05. All of the statistical analyses were performed using SPSS, version 16.0 (SPSS, Inc., Chicago, IL, USA).

## References

[1] Surveillance, Epidemiology, and End Results (SEER) Program Public-Use CD-ROM (1973–1997).

[2] Walker RA, Lees E, Webb MB, Dearing SJ (1996). Breast carcinomas occurring in young women (<35 years) are different. Br J Cancer.

[3] Adami HO, Malker B, Holmberg L, Persson I, Stone B (1986). The relation between survival and age at diagnosis in breast cancer. N Engl J Med.

[4] Chung M, Chang HR, Bland KI, Wanebo HJ (1996). Younger women with breast carcinoma have a poorer prognosis than older women. Cancer.

[5] Winchester DP, Osteen RT, Menck HR (1996). The National Cancer Data Base report on breast carcinoma characteristics and outcome in relation to age. Cancer.

[6] Kwon JH, Kim YJ, Lee KW, Oh DY, Park SY, Kim JH, Chie EK, Kim SW, Im SA, Kim IA, Kim TY, Park IA, Noh DY, Bang YJ, Ha SW (2010). Triple negativity and young age as prognostic factors in lymph node-negative invasive ductal carcinoma of 1 cm or less. BMC Cancer.

[7] Theriault RL, Litton JK, Mittendorf EA, Chen H, Meric-Bernstam F, Chavez-Macgregor M, Morrow PK, Woodward WA, Sahin A, Hortobagyi GN, Gonzalez-Angulo AM (2011). Age and survival estimates in patients who have node-negative T1ab breast cancer by breast cancer Subtype. Clin Breast Cancer.

[8] Holli K, Isola J (1997). Effect of age on the survival of breast cancer patients. Eur J Cancer.

[9] Rakha EA, El-Sayed ME, Green AR, Paish EC, Powe DG, Gee J, Nicholson RI, Lee AH, Robertson JF, Ellis IO (2007). Biologic and clinical characteristics of breast cancer with single hormone receptor positive phenotype. J Clin Oncol.

[10] Althuis MD, Fergenbaum JH, Garcia-Closas M, Brinton LA, Madigan MP, Sherman ME (2004). Etiology of hormone receptor-defined breast cancer: a systematic review of the literature. Cancer Epidemiol Biomarkers Prev.

[11] Tang LC, Yin WJ, Di GH, Shen ZZ, Shao ZM (2010). Unfavourable clinicopathologic features and low response rate to systemic adjuvant therapy: results with regard to poor survival in young Chinese breast cancer patients. Breast Cancer Res Treat.

[12] Lord SJ, Bernstein L, Johnson KA, Malone KE, McDonald JA, Marchbanks PA, Simon MS, Strom BL, Press MF, Folger SG, Burkman RT, Deapen D, Spirtas R, Ursin G (2008). Breast cancer risk and hormone receptor status in older women by parity, age of first birth, and breastfeeding: a case–control study. Cancer Epidemiol Biomarkers Prev.

[13] Phipps AI, Chlebowski RT, Prentice R, McTiernan A, Wactawski-Wende J, Kuller LH, Adams-Campbell LL, Lane D, Stefanick ML, Vitolins M, Kabat GC, Rohan TE, Li CI (2011). Reproductive history and oral contraceptive use in relation to risk of triple-negative breast cancer. J Natl Cancer Inst.

[14] Setiawan VW, Monroe KR, Wilkens LR, Kolonel LN, Pike MC, Henderson BE (2009). Breast cancer risk factors defined by estrogen and progesterone receptor status: the multiethnic cohort study. Am J Epidemiol.

[15] Hankinson SE, Tamimi R, Hunter D, Adami H, Hunter D, Trichopoulos D (2008). Breast cancer. In Textbook of Cancer Epidemiology.

[16] Rosenberg L, Boggs DA, Wise LA, Adams-Campbell LL, Palmer JR (2010). Oral contraceptive use and estrogen/progesterone receptor-negative breast cancer among African American women. Cancer Epidemiol Biomarkers Prev.

[17] Hilton HN, Doan TB, Graham JD, Oakes SR, Silvestri A, Santucci N, Kantimm S, Huschtscha LI, Ormandy CJ, Funder JW, Simpson ER, Kuczek ES, Leedman PJ, Tilley WD, Fuller PJ, Muscat GE (2014). Acquired convergence of hormone signaling in breast cancer: ER and PR transition from functionally distinct in normal breast to predictors of metastatic disease. Oncotarget.

[18] Jin X, Jiang YZ, Chen S, Yu KD, Shao ZM, Di GH (2015). Prognostic value of receptor conversion after neoadjuvant chemotherapy in breast cancer patients: a prospective observational study. Oncotarget.

